# Pharmacokinetics, SAfety/tolerability, and EFficacy of high-dose RIFampicin in tuberculosis-HIV co-infected patients on efavirenz- or dolutegravir-based antiretroviral therapy: study protocol for an open-label, phase II clinical trial (SAEFRIF)

**DOI:** 10.1186/s13063-020-4132-7

**Published:** 2020-02-13

**Authors:** Ruth Nabisere, Joseph Musaazi, Paolo Denti, Florence Aber, Mohammed Lamorde, Kelly E. Dooley, Rob Aarnoutse, Derek J. Sloan, Christine Sekaggya-Wiltshire

**Affiliations:** 1 Infectious Diseases Institute, Makerere University College of Health Sciences, Kampala, Uganda; 2 Division of Clinical Pharmacology, Department of Medicine, University of Cape Town, Cape Town, South Africa; 3 Division of Clinical Pharmacology, Department of Medicine, Johns Hopkins University School of Medicine, Baltimore, MD USA; 4 Radbound University, Radbound, Netherlands; 5 Division of Infection and Global Health, School of Medicine, University of St. Andrews, St. Andrews, Scotland

**Keywords:** Rifamycins, Antiretrovirals, HIV, Pharmacokinetics, Tuberculosis

## Abstract

**Background:**

Tuberculosis (TB) is a significant public health problem that causes substantial morbidity and mortality. Current first-line anti-TB chemotherapy, although very effective, has limitations including long-treatment duration with a possibility of non-adherence, drug interactions, and toxicities. Dose escalation of rifampicin, an important drug within the regimen, has been proposed as a potential route to higher treatment efficacy with shorter duration and some studies have suggested that dose escalation is safe; however, these have almost entirely been conducted among human immunodeficiency (HIV)-negative TB patients. TB-HIV co-infected patients on antiretroviral therapy (ART) are at increased risk of drug-drug interactions and drug-related toxicities. This study aims to determine the safety of higher doses of rifampicin and its effect on the pharmacokinetics of efavirenz (EFV) and dolutegravir (DTG) in TB-HIV co-infected patients.

**Methods:**

This study is a randomized, open-label, phase IIb clinical trial among TB-HIV infected adult outpatients attending an HIV clinic in Kampala, Uganda. Patients newly diagnosed with TB will be randomized to either standard-dose or high-dose rifampicin (35 mg/kg) alongside standard TB treatment. ART-naïve patients will be randomly assigned to first-line ART regimens (DTG or EFV). Those who are already on ART (DTG or EFV) at enrollment will be continued on the same ART regimen but with dose adjustment of DTG to twice daily dosing. Participants will be followed every 2 weeks with assessment for toxicities at each visit and measurement of drug concentrations at week 6. At the end of intensive-phase therapy (8 weeks), all participants will be initiated on continuation-phase treatment using standard-dose rifampicin and isoniazid.

**Discussion:**

This study should avail us with evidence about the effect of higher doses of rifampicin on the pharmacokinetics of EFV and DTG among TB-HIV co-infected patients. The trial should also help us to understand safety concerns of high-dose rifampicin among this vulnerable cohort.

**Trial registration:**

ClinicalTrials.gov, ID: NCT03982277. Registered retrospectively on 11 June 2019.

## Background

Tuberculosis (TB) is a significant public health burden that causes substantial morbidity and mortality. Globally, approximately 10 million people developed TB in 2017, 9% of whom were people living with human immunodeficiency (HIV) (72% of these residing in Africa). Eighty-seven percent of all the world’s TB cases were accounted for by the 30 high-burden TB countries [1].

Rifampicin is a key component of current first-line TB treatment. It contributes to rapid bactericidal activity during the first few days of TB treatment, and it has a sterilizing effect that lasts throughout the treatment course [2]. Even with good adherence to therapy inter-individual plasma exposure to rifampicin is known to be variable. Low exposure has been associated with drug resistance, treatment failure, and delayed bacterial clearance from sputum [3–5]. Higher doses of rifampicin have been associated with a faster drop in bacterial load over time. Shorter treatment regimens including high-dose rifampicin are currently being investigated [6]. Several studies have suggested that dose escalation is safe; however, these have almost entirely been conducted among HIV-negative TB patients, or TB-HIV co-infected patients without severe immunosuppression who were not yet receiving antiretroviral therapy (ART) [7, 8]. TB-HIV co-infected patients on multiple additional drugs, including ART, are at increased risk of drug-drug interactions and drug-related toxicities, including hepatotoxicity [9, 10].

Rifampicin induces a plethora of metabolic processes through the pregnane-x receptor [11], including cytochrome P450A, thus leading to several drug-drug interactions. While a few small studies suggest that maximal induction already occurs at relatively low doses of rifampicin, the dose that achieves the maximal induction effect is still unknown.

Rifampicin induces CYP2B6 (responsible for efavirenz (EFV) metabolism) and UGT1A1, and CYP3A4 (responsible for dolutegravir (DTG) metabolism). Although earlier studies demonstrated that the standard-dose rifampicin does not significantly decrease EFV concentrations, (which in some studies has been attributed to the inhibitory effect of isoniazid on cytochrome P450 2A6 [12, 13]), and doubling the dose of DTG overcomes the enzyme-induction effect, the consequences of administering higher doses of rifampicin for HIV-TB co-infected patients on ART requires specific, careful evaluation [14, 15].

Rifampicin-dose escalation may help to improve TB treatment outcomes. However, there is almost no information on the enzyme-induction effect of high-dose rifampicin on EFV and DTG and a paucity of data on the safety of higher rifampicin doses in HIV-infected patients on ART. If higher doses of rifampicin are to be considered for routine TB treatment in African countries, such data will be critical for the high number of HIV co-infected persons. The aim of this study is to determine the effect of higher-dose rifampicin on the pharmacokinetics (PK) of EFV and DTG in TB-HIV co-infected patients, and to establish whether higher-dose rifampicin is safe for HIV-infected patients with TB.

## Methods

### Study site

This study is being conducted at the integrated TB-HIV Outpatient Clinic of the Infectious Diseases Institute Makerere University College of Health Sciences in Kampala, Uganda, which is an urban outpatient HIV clinic that provides care to 200 patients infected with TB annually.

### Study design

This is a randomized, open-label, phase IIb clinical trial of HIV-infected patients newly diagnosed with TB.

### Study population

Patients are enrolled if they fulfill the following inclusion criteria: **(**1) adults of age ≥ 18 years, (2) confirmed HIV-1 infection, (3) already started on EFV- or DTG-based ART or planned to start on ART (national first-line regimens are DTG- or EFV-based), and (4) diagnosed with TB and due to initiate rifampicin-containing therapy.

Patients will be excluded if they meet any one of the following criteria: (1) have rifampicin-resistant TB identified by baseline Xpert *Mycobacterium tuberculosis*/rifampicin (MTB/RIF), (2) pregnant women, (3) women of reproductive age on DTG who decline the use of effective contraception methods (in particular: intrauterine device or condoms and dual contraception for those on hormonal methods), (4) decompensated liver disease and/or aminotransferases > 5 x upper limit of normal (ULN), and (5) glomerular filtration rate (GFR) < 50 ml/min.

### Study objectives

#### Primary objective

1. To determine the effect of a high dose of rifampicin (35 mg/kg orally) on the PK of first-line antiretroviral drugs (EFV and DTG) in TB-HIV co-infected patients on TB treatment

#### Secondary objectives

2. To investigate the safety/tolerability of higher doses of rifampicin in TB-HIV co-infected patients on TB treatment and first-line antiretroviral drugs (EFV and DTG)
3. To determine if TB-HIV co-infected patients on higher doses of rifampicin are more likely to have negative sputum cultures by the end of the intensive phase of TB treatment than patients on standard-dose rifampicin
4. To explore the relationships between exposure to rifampicin, EFV, and DTG and the tolerability and efficacy of these drugs (PK-pharmacodynamic (PD) analysis)

### Study hypotheses

- *Patients on a high dose of rifampicin will have a minimal decrease in exposure to EFV and DTG compared to those on standard-dose rifampicin*
- *A higher proportion of patients on first-line ART and high-dose rifampicin will experience hepatotoxicity compared to those on standard-dose rifampicin, but this increase will be modest, and hepatotoxicity events will be mostly low-grade*
- *A higher proportion of patients on high-dose rifampicin will have negative cultures at week 8 compared to those on standard-dose rifampicin*
- *Antiretroviral response will be similar among patients who use standard- or high-dose rifampicin as part of their TB treatment*

### TB diagnosis

TB is diagnosed using chest x-ray, sputum florescent microscopy or Xpert MTB/RIF, and a clinical history that includes any one of the following symptoms: cough, fever, weight loss, and drenching night sweats. Patients with confirmed TB (positive Xpert, positive urinary lipoarabinomannan (LAM), or TB adenitis confirmed by Ziehl-Neelsen (ZN) staining or histopathology) are included in the study.

### Intervention

Participants are randomized to either standard-dose (10 mg/kg) or high-dose (35 mg/kg) rifampicin. All other anti-TB drugs (isoniazid, ethambutol, and pyrazinamide (HEZ)) are given at their standard dose for weight bands using Fixed Dose Combination (FDC) tablets supplied by the National TB Control Program. The patients on high-dose rifampicin have their additional doses supplemented by rifampicin capsules.

ART-naïve patients are randomly assigned to first-line ART regimens (DTG 50 mg twice daily or EFV 600 mg once daily) which is initiated 2 weeks into TB treatment. Those who are already on ART at enrollment continue the same ART regimen; however, participants on DTG are switched to twice-daily DTG while they are on rifampicin. EFV/DTG is given in combination with tenofovir, zidovudine, or abacavir plus either lamivudine or emtricitabine. Most patients are given tenofovir/lamivudine as backbone therapy because it is the preferred first-line backbone therapy nationally and they are only offered zidovudine or abacavir if there are contraindications to tenofovir. Viral-load measurements are performed at baseline (for patients on ART at baseline) and at the week-24 visit (6 months after initiation of ART).

Participants are followed up every 2 weeks for study procedures including assessment for toxicities (Figs. 2 and 3 in Appendix). At the end of the intensive phase (8 weeks), all participants will be initiated on the continuation phase using standard-dose rifampicin and isoniazid. Adherence is monitored using pill counts and self-report.

### Randomization process

**Fig. 1:**
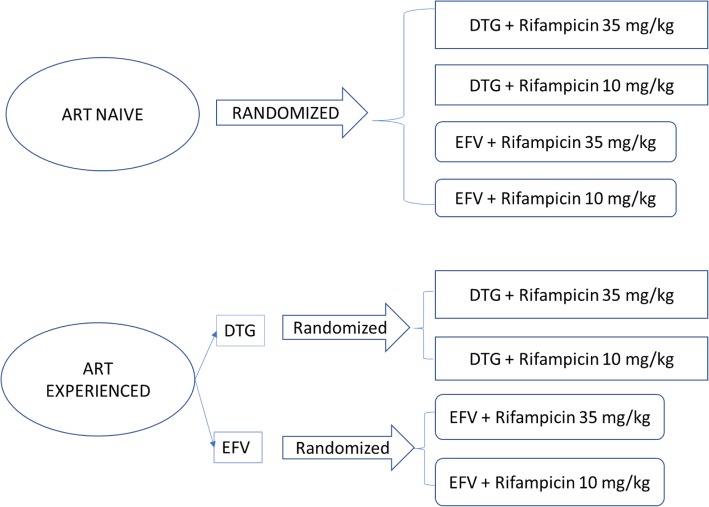
Diagram showing the randomization process

**Fig. 2:**
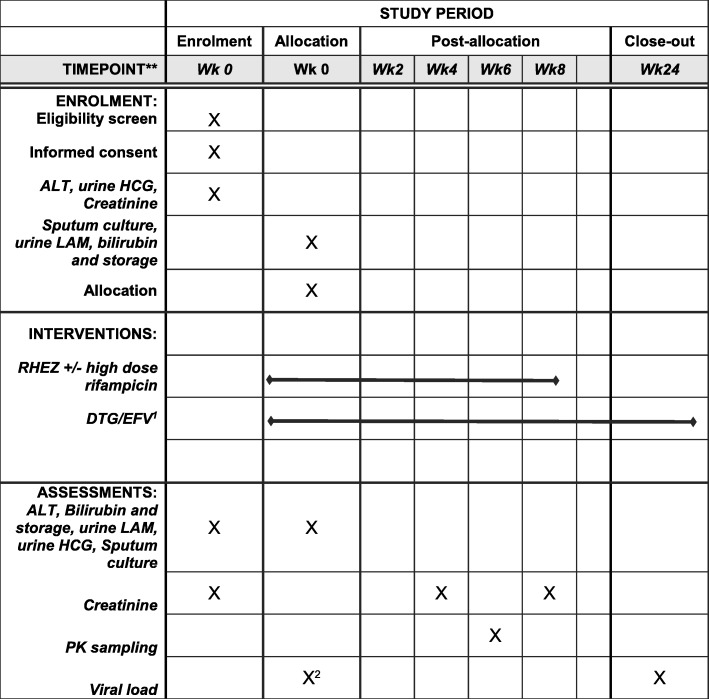
Schedule of enrollment, interventions, and assessments

**Fig. 3:**
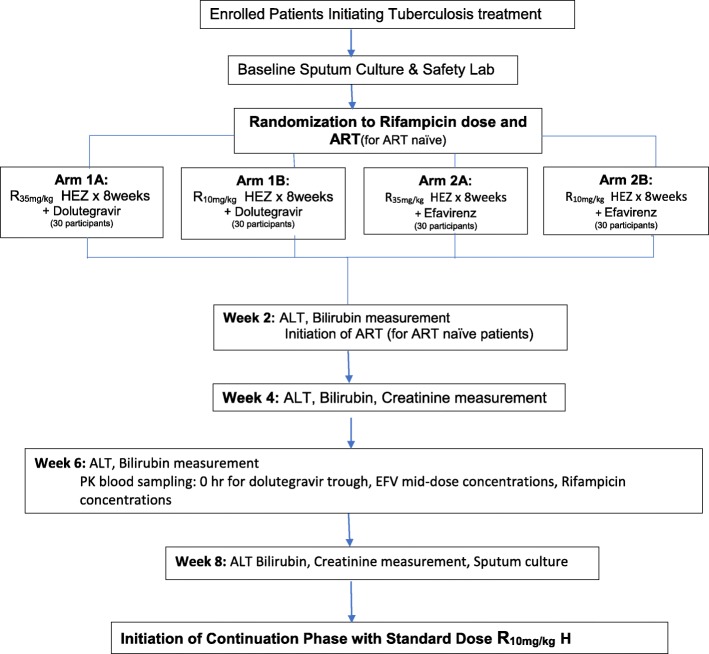
Study schema

Randomization is done by the trial pharmacist using a computer-generated randomization code that assigns participants to four treatment allocation groups (refer to Fig. 1 below), with 30 participants in each group: (1) high-dose rifampicin and DTG, (2) standard-dose rifampicin and DTG,.(3) high-dose rifampicin and EFV, and (4) standard-dose rifampicin and EFV. Details of a patient’s allocation is then notified to the study coordinator immediately. This is an open-label study; however, participants and data analysts are blinded to the treatment arm through the use of anonymized codes during sample collection and analysis.

### Pharmacokinetic measurements

Blood sampling for PK analysis is performed 6 weeks after initiation of TB treatment. On the study visit, participants come to the clinic in the morning after fasting overnight and observed intake of anti-TB drugs and the morning dose of DTG occurs. Blood samples to measure rifampicin and dolutegravir concentrations are drawn prior to drug intake (0 h), and at 1, 2, 4, and 8 h following observed intake of the anti-TB drugs. Mid-dose EFV concentrations are measured at 0 h.

The blood samples are immediately placed in a dark cooler and transported to the laboratory within 30 min of collection, after which samples are centrifuged, aliquoted, batched, and frozen at − 80 °C until quantification of drug concentrations is done. Quantification of the drug concentrations (rifampicin, EFV, DTG) will be performed using pre-established, validated, high-performance liquid chromatography (HPLC) assays.

### Microbiological assessment

Sputum samples are collected from patients with pulmonary TB for culture on liquid medium (Mycobacteria Growth Indicator Tube (MGIT) and solid medium (BACTEC) at baseline and 8 weeks of anti-TB treatment. Additional samples will be collected for storage every 2 weeks during the first 2 months of TB treatment, for storage for future cultures using mycobacterial-load assay.

### Adverse event assessment

Participants are assessed for adverse events on every study visit using direct questioning, physical examination, and laboratory parameters; alanine transferase (ALT) and bilirubin (2 weekly) and creatinine (4 weekly). The severity of liver toxicity is graded according to the National Institutes of Health DAIDS [14]. Study medication will be stopped when either of the following occurs: (1) symptoms of hepatitis (for example, abdominal pain, nausea, vomiting, malaise) and ALT measurement ≥ 3 times the ULN, (2) ALT is ≥ 5 times the ULN regardless of symptoms, and (3) any toxicity that, in the investigator’s judgment, is due to study drug and requires interruption of treatment with the causative study drug. Causality assessment for all adverse events will be performed and classified as either definite, probable, possible, unlikely or unrelated. serious adverse events will be reported to the sponsor within 24 h and the regulatory authorities within 7 days.

Anti-TB medication, under the standard of care, will be reintroduced at their standard doses when ALT is within the normal range. All serious adverse events are followed up until resolution even after participants are withdrawn from study.

There is no anticipated harm and compensation for trial participation.

Additional blood samples will be stored at baseline, weeks 2, 4, 6, and 8 for validation of new biomarkers used for early detection of drug-induced liver injury.

## Data analysis

### Sample size calculation

We applied a sample size calculation using coefficient of variation and percentage change in geometric mean of EFV mid-dose concentrations. This study is powered on the primary endpoint of mid-dose EFV concentrations. We varied the difference in the concentrations among the different dose arms (high-dose arm vs. standard-dose arm) between 5 and 30%, which is close to the bioequivalence accepted by the Food and Drug Authority. With a power of 80%, a difference in mid-dose EFV concentrations of 30% and assuming loss to follow-up of 25%, we would need a sample size of 120 participants (30 patients in each group). Patients are actively referred from city council hospitals in order to achieve our sample size within the target recruitment period of 1 year.

Patient retention: the study team is responsible for tracking participants using phone calls, and where possible, home visits, to ensure retention rate. Locator information is collected and updated at each visit where necessary. Outcome data will be collected from patients who deviate or discontinue assigned treatment on their clinic visits or through phone interviews.

### Analysis of endpoints

#### Primary objective (drug interactions)

We will compare the EFV mid-dose concentrations and the trough concentrations of DTG in patients on 35 mg/kg and 10 mg/kg doses using an independent samples *T* test on logarithmically transformed PK measurements or a Wilcoxon rank sum test on untransformed data.

### Pharmacokinetic data

In addition, we will apply a population PK-modeling approach for PK data. We will develop population models to describe the PK parameters of rifampicin, EFV, and DTG and the between-subject and between-occasion variability in these parameters. The models will be based on previously developed PK models that will be adapted by fitting them to the data collected in the study.

We will compare the proportions of patients who develop liver toxicity and the severity/grade of liver toxicity among patients on the different doses of rifampicin using the chi-squared test. We will determine the association between each rifampicin dose and hepatotoxicity using Cox regression.

We will estimate the proportion of patients who remain culture-positive after 8 weeks of anti-TB treatment in each treatment arm and compare them using the chi square test. Using non-linear mixed effects modeling, we will develop PK-PD models to determine the relationship between PK exposure and PD response including virological suppression, toxicities, and bacteriological response.

Analysis of primary endpoint will be performed per protocol whereas intention-to-treat analysis will be performed as sensitivity analysis. Missing data on drug concentration will be imputed using multiple imputation when performing sensitivity analysis.

### Quality control and assurance

During the study, periodic monitoring is conducted to ensure that the protocol and International Conference on Harmonization-Good Clinical Practice (ICH-GCP) principles are being followed. Additionally, the study site may be subject to review by the Institutional Review Board and the regulatory authorities.

### Data handling and record retention

De-linked clinical and demographic data is collected on Case Report Forms (CRF) and kept in a locked cabinet with access granted only to authorized study staff. Data from the CRFs is stored via Datafax, which reads the data using intelligent character recognition and enters the data into a secure server at the Infectious Diseases Institute. Data will be securely stored for a minimum of 20 years according to the local National Drug Authority regulations for clinical trials.

The independent Data Safety and Monitoring Board (DSMB) will make recommendations concerning the study to the Trial Steering Committee. The DSMB is chaired by Dr. Catriona Waitt a clinical pharmacologist from the University of Liverpool and based at the Infectious Diseases Institute, Kampala, with experience in dose-finding PK studies, Dr. Agnes Kiragga, a senior statistician at the Infectious Diseases Institute with experience in HIV clinical trials and cohort studies, Dr. Marta Boffito, the clinical research lead at St Stephen’s AIDS Trust in the U.K, who has extensive expertise in complex pharmacological issues, and Dr. Eric Decloedt, a registrar in clinical pharmacology and a senior lecturer and researcher at Stellenbosch University in South Africa. The DSMB is independent from the sponsor and has no competing interests. The first interim analysis will be conducted when 10% of the participants complete the study medication. Trial monitoring is conducted independent of the investigators by internal monitors at the Infectious Diseases Institute. The Trial Steering Committee is composed of Dr. Stella Zawedde (chair), a representative from the National Tuberculosis and Leprosy Program in Uganda, Dr. Pauline Byakika-Kibwika and Associate Professor David Meya, both infectious disease specialists at the Department of Internal Medicine of Makerere University, Dr. Susan Adakun, head of the Mulago National Referral Hospital Tuberculosis Unit, and Elizabeth Tindyebwa, a lay representative from the Friends’ Council (patient group at IDI). Regular discussions are held over email or conference calls when required but at least once a year. The Trial Management Group meets weekly and is responsible for the day-to-day study activities. It is composed of the Dr. Christine Sekaggya (principal investigator who provides overall oversight), Dr. Derek Sloan, and Dr. Mohammed Lamorde (co-investigators), Dr. Ruth Nabisere (trial manager), Dr. Brian Otalo (study physician), Florence Aber, Juliet Nampala (study nurses), Joseph Musaazi (statistician), and Hamza Mayanja (pharmacist and person responsible for randomization).

## Ethical issues

All precautions are being made to ensure the safety of patient data and identifying information. All patients provide written informed consent for participation in the study and the storage of biological samples including blood, urine, and sputum for ancillary studies, a process conducted by the study nurse and study coordinator.

Ethical approval for this study was sought from the Joint Clinical Research Council (*JC2218*), the National Drug Authority, and the Uganda National Council for Science and Technology.

The study is being conducted in accordance with the general principles set forth in the International Ethical Guidelines for Biomedical Research Involving Human Subjects and the Declaration of Helsinki and Good Clinical Practice guidelines. The study is registered at ClinicalTrials.gov, ID: NCT03982277. and has been reported according to SPIRIT guidelines.

All protocol amendments will be first submitted to the responsible regulatory bodies before implementation. Protocol deviations will be reported to the regulatory bodies within 7 days of site notification and recorded in a protocol deviation log kept in the regulatory binder. The protocol will also be updated at ClinicalTrials.gov.

## Dissemination of results

Study results will be published in peer-reviewed journals with open access. We will also present our findings to the National TB Program, local, regional, and international conferences.

## Discussion

This study is one of the few trials to investigate rifampicin at 35 mg/kg, and the first ever to do so in a higher-risk population (HIV-infected patients). It should provide crucial evidence about the PK and the safety of co-administration of EFV and DTG with 35 mg/kg of rifampicin in a TB-HIV co-infected population. We are including a population of patients with severe immunosuppression that will be representative of the high proportion of TB patients, who are also co-infected with HIV.

This study should be able to demonstrate whether higher doses of rifampicin can be safely used in HIV-positive patients on ART, and whether this has an impact of their treatment outcomes with faster mycobacterial-load clearance.

With the stored samples from this population, we will conduct a pharmacogenomic analysis to understand the role of genetic polymorphisms in the PK of the investigated drugs and evaluate new molecular assays that can be used for early detection of hepatotoxicity.

However, the investigators also acknowledge some of the anticipated challenges and/or limitations of this study which include:

- There is need for more vigilant safety monitoring coupled with a very strong Data and Safety Monitoring Board due to the anticipated toxicities and drug-drug interactions that we might experience in the trial
- Secondly, there is no blinding in this clinical trial, hence it is hard to completely rule out the effect of bias in interpreting possible toxicities that are not backed by objective laboratory parameters
- Currently, because of the test-and-treat recommendation, some patients are already on ART and, therefore, randomization to ART for some patients is not possible

Despite these, we believe that this study will demonstrate that early phase trials can be successfully conducted in resource-constrained settings where the highest burden of the dual epidemic lies.

## Trial status

The study, with protocol version 3, 17 December 2018, began enrollment on 30 April 2019 and had recruited 37 participants by 18 October 2019. The expected end of enrollment is 30 December 2020.

### Supplementary information

**Additional file 1.** Standard Protocol Items*:* Recommendations for Interventional Trials (SPIRIT) 2013 Checklist: recommended items to address in a clinical trial protocol and related documents.

## Data Availability

Any data required to support the protocol can be supplied on request.

## Abbreviations

ART: Antiretroviral therapy
DTG: Dolutegravir
EDCTP: European and Development Countries Clinical Trial Program
EFV: Efavirenz
HIV: Human immunodeficiency virus
HPLC: High-performance liquid chromatography
MGIT: Mycobacteria Growth Indicator Tube
MTB/Rif: *Mycobacterium tuberculosis*/rifampicin
PK: Pharmacokinetics
PK-PD: Pharmacokinetics/pharmacodynamics
TB: Tuberculosis
ULN: Upper limit of normal
